# Transplantation in resource-limited setting: using HIV-positive donors for HIV-positive patients 

**DOI:** 10.5414/CNP83S0039

**Published:** 2015-02-23

**Authors:** Elmi Muller

**Affiliations:** Groote Schuur Hospital, University of Cape Town, South Africa

**Keywords:** transplantation, HIV-positive donors, HIV-positive recipients

## Abstract

Abstract. Background: A HIV positive-to-positive program was started in South Africa in 2008. The program was started because dialysis is not freely available to everyone, but severely limited and only available to a selected group of patients. Patients and Methods: Between September 2008 and March 2015, 29 patients were transplanted from HIV-positive brain-dead donors at Groote Schuur Hospital transplant team. Donors were either naïve to anti-retroviral therapy or on first line therapy. The recipients were selected to have undetectable plasma HIV type 1 RNA levels and be on a stable antiretroviral regimen. CD4+ T-cell counts of at least 200/mm^3^ in last 6 months prior to transplant, with no previous serious opportunistic infections. Results: Survivors in the study were followed for a median of 2.4 years. The rate of patient survival was 84% at 1 year and 74% at 5 years. The corresponding graft survival rate was 93% and 84%. Conclusion: Using HIV-positive donors might resolve some of the problems we are experiencing in getting enough donors for our patients wit ESRD. In the USA the HOPE act was accepted in 2014 and this might now also impact on the use of HIV positive donors elsewhere in the world.

## Introduction 

South Africa currently offers dialysis and transplantation as a treatment option for patients with end stage renal disease (ESRD). However, dialysis is not freely available to everyone, but severely limited and only available to a selected group of patients. This means that patients get assessed when they present with ESRD and they only get accepted onto a dialysis program if they fulfil certain criteria. These criteria are criteria to assess the patient’s medical fitness in general as well as social criteria to assess whether the patient will be compliant with follow-up. In most state hospitals, patients will only be accepted onto a dialysis program if they are also fit to receive a transplant in the long run. The idea is that dialysis programs should naturally feed into transplant programs. Therefore, a patient who is not a suitable transplant candidate will normally be turned down for dialysis. 

## Background 

In 2008, when the HIV positive-to-positive program started, patients with ESRD and HIV would be turned down for dialysis. The reason was that they were seen as unfit for transplantation and therefore not suitable dialysis patients. This meant that anybody with HIV and ESRD was doomed to die. This situation remained unchallenged for a number of years, especially as the rollout of antiretroviral therapy was quite slow in the state sector. 

The HIV population with ESRD started to grow dramatically in 2008 in South Africa. A major problem with having HIV is that a large percentage of these people will develop HIV-associated nephropathy (HIVAN). Histologically, HIVAN is a collapsing form of focal sclerosing glomerulosclerosis (FSGS), which can be distinguished from idiopathic FSGS by the presence of microcystic tubular dilatation and interstitial inflammation [1]. 

Modelling the incidence of ESRD and HIV in Sub Sahara Africa we know that we have at least 20.9 million people over the age of 15 years with HIV-related chronic kidney disease (CKD), according to UNAIDS prevalence statistics. It is estimated that ~ 60% of these people are treated with anti-retroviral therapy. 

## Incidence of kidney disease in HIV positive patients 

Whether or not an individual is receiving treatment influences the rate of mortality in the HIV-CKD population, and also affects the rate of progression to ESRD in the population with HIVAN. Although the proportion of HIV-CKD that is attributable to HIVAN is variable, biopsy studies have reported that ~ 30% of CKD in the HIV positive South African population is attributable to HIVAN, and that treatment reduces the risk of developing ESKD in HIVAN by ~ 60% [2, 3]. 

Because of very high HIV rates in the country, more and more HIV-positive brain-dead donors presented to the Groote Schuur Hospital Transplant team. These donors were mostly brain-dead people who were worked up for organ donation (after consent was obtained from the family) and who then turned out to be HIV positive. In 2008 it made sense to try and marry this supply of donors with the group of HIV positive patients without any treatment options in the country. 

## Problems with using HIV positive donors 

Concerns about a second viral strain remained a problem. In the literature the outcomes and reports of HIV-positive patients with super-infections are difficult to interpret as there are a lot of methodological difficulties which often yield conflicting results [4, 5]. When a patient with low viral load gets exposed to a second viral strain, a superinfecting strain may be detectable for only a short period of time. Viral fitness and the ability of a viral strain to replicate effectively in a given environment, may play a role to determine whether the two different strains will eventually become undetectable in standard resistance tests or whether outgrowth of a different virus from the baseline or whether a novel recombinant virus will become detectable [4]. 

## South African situation 

In South Africa we have a unique situation in view of the fact that we have low antiretroviral therapy resistance rates [6, 7, 8, 9]. Most patients who failed second-line ART in South Africa, have wild-type virus and resistance rates remain less than 5% in our HIV population. So in our setting the issues transplanting HIV-positive patients are mostly that they have very high rejection rates, that they need powerful and expensive immunosuppression as these patients have a dysregulated immunosystem rather than a suppressed one. They also have a high infection risk as opportunistic infections are more common in immunosuppressed and HIV-positive patients, and in Africa opportunistic infection remains a major reason why transplant patients might run into trouble [10]. 

## Exclusion and inclusion criteria 

The HIV positive-to-positive program in Cape Town is a deceased donor program where the donors are HIV positive and selected according to the following criteria:

- ART-naïve donor
- If on ART, must be on first line treatment with no resistance
- Normal serum creatinine
- No proteinuria
- Protocol biopsy on reperfusion of kidney

No donor with the following problems is used:

- Active viral/fungal/parasitic infection
- Malignancies
- Sepsis
- Possible HIVAN present

Recipients are selected to have undetectable plasma HIV type 1 RNA levels and be on a stable antiretroviral regimen. CD4+ T-cell counts of at least 200/mm^3^ in last 6 months prior to transplant, with no previous serious opportunistic infections. If a patient had previous tuberculosis infection, it must be fully treated. 

## Results

In the study we have enrolled 29 patients over the last 5 years. Five patients died after transplant. The reasons for death were myocardial infarction, lung squamous cell cancer, pancreatitis with a duodenal perforation, disseminated *Aspergillosis* and *Klebsiella Pneumonia* sepsis. Two patients lost their grafts in the 1^st^ week after transplantation: 1 with venous thrombosis of the graft and 1 with acute severe rejection within 1 week after transplantation. A 3^rd^ patient lost her graft with chronic vascular rejection and fibrosis of the graft 2 years after her transplant. The risk of rejection in this patient population group is higher than expected in HIV-negative patients. In the Cape Town study, rejection took place on 8 occasions in 5 of the patients, which gives an acute rejection rate of 18%. This happened despite induction therapy with thymoglobuline. A dysregulated immune response might be the reason for high rejection rates despite potent immunosuppression, and similar high rejection episodes were reported in the NIH study using HIV-negative donors for HIV-positive recipients [11]. 

Using HIV-positive donors might resolve some of the problems we are experiencing in getting enough donors for our patients with ESRD. In the USA, the HOPE act was accepted in 2014 and this might now also impact on the use of HIV-positive donors elsewhere in the world. 

## Conflict of interest 

None. 
